# Evaluating Operation Good Food & Beverages, a Black Youth-Driven Public Advocacy Campaign

**DOI:** 10.1007/s40615-024-02150-6

**Published:** 2024-09-03

**Authors:** Matthew D. Kearney, Tiffany M. Eaton, Megan Grabill, Siani Anderson, Shiriki Kumanyika

**Affiliations:** 1 Department of Family Medicine and Community Health, University of Pennsylvania Perelman School of Medicine, Penn Presbyterian Medical Center, 51 N. 39th Street, Philadelphia, PA USA; 2 Drexel University Dornsife School of Public Health, Department of Community Health and Prevention, Philadelphia, PA USA; 3 Department of Biostatistics, Epidemiology, and Informatics, University of Pennsylvania Perelman School of Medicine, Philadelphia, PA USA

**Keywords:** Black youth, Food and beverage marketing, Social media, Health communication

## Abstract

**Background:**

Food and beverage (F&B) marketing practices that contradict health guidelines are particularly concerning for children and adolescents, who are developmentally more susceptible than adults to persuasive advertising and to Black communities, due to ethnically-targeted marketing, contributing to higher rates of obesity and other diet-related chronic diseases. Accordingly, here we evaluated Operation Good Food and Beverages (OGF&B), an online social marketing campaign calling for shifting toward more marketing of healthier F&B to Black youth and Black communities.

**Methods:**

OGF&B was developed and implemented by a multidisciplinary team of academic, advocacy, and advertising partners and active for four months in 2022 during the COVID-19 pandemic. Primary campaign components were social media content (e.g., TikTok, Instagram), and an informational website with a signable petition and a social media toolkit. Our mixed-methods evaluation used qualitative data to contextualize quantitative metrics like online impressions, website visits, and petition signatures. Qualitative data consisted of analysis of social media content and thematic elements from 15 interviews with campaign advisors, youth consultants, and influencers.

**Results:**

The campaign achieved 3,148,869 impressions, 3,799 unique website visits, and 1,077 petition signatures. Instagram Reels and content featuring people had higher engagement. Instagram Reels received more likes than static posts or TikTok videos. Interviewees who participated mentioned personal values and community welfare as key motivations. Social media influencers who declined participation noted time constraints and lack of compensation as barriers.

**Conclusion:**

Despite pandemic-related restrictions that precluded in-person engagement, this brief campaign implementation period provided useful insights for leveraging OGF&B or similar campaigns.

## Introduction

Food and beverage (F&B) marketing practices are in striking contradiction to the types of foods and beverages recommended in health guidelines, and there is wide agreement, within the USA and globally, on the need for change in this realm [1, 2]. Advertising and promotion of products high in calories, sugar, fat, and salt are ubiquitous compared to relatively limited marketing of fruits and vegetables or healthier versions of packaged foods [3]. Efforts to change this picture have included taxing F&B products high in fat, salt, and sugar to discourage their consumption, discounts to incentivize fruit and vegetable purchases for participants in federal nutrition assistance programs, and strict nutrition standards that improve the quality of foods that can be served in school meals [4–6]. However, given that the least healthy products are typically the most profitable [7], a stronger regulatory climate will likely be needed to achieve the type and scope of changes needed [8]. With the ultimate goals of increasing availability and promotion of healthier products, consumer advocacy across diverse media and communication channels has been and will continue to be an important component of support for relevant regulatory changes.

The various types of food advertising and promotions that target children and adolescents are of particular concern because of the importance of healthy eating patterns for growth combined with developmental issues that make youth more susceptible to the persuasive content of advertisements [9–11]. For example, a younger child may be more susceptible because they lack the ability to identify advertising as having a persuasive intent and resist persuasive advertising, while an adolescent may recognize persuasive content yet still be susceptible because they want to fit in with their peers [11, 12]. The rise in obesity prevalence among children and adolescents has been a prominent public health concern for more than two decades, with no consistent evidence that rates are stabilizing or decreasing [13–16]. The substantial targeting of youth with marketing of high calorie, nutrient poor F&B (e.g., on television, through various digital techniques, and in public spaces) is identified as an important contributor to obesity among youth and their later risks of obesity-related chronic diseases [17, 18]. In synergy with advertisements, the advertised F&B products are visible and readily available in environments frequented by youth, including neighborhood stores, supermarkets, and chain restaurants [19–22]. Industry’s voluntary commitments (i.e., “self-regulation”) to offer healthier options to children under age 12 have shown limited progress but still with major opportunities for improvement and very few companies have extended commitments to include youth over age 12 [23–26].

Of particular relevance here is the evidence that these challenges are exacerbated by the additional layer of targeted marketing on the basis of ethnicity. Ethnic targeting of F&B marketing is well documented as a potential contributor to obesity and chronic disease risks of Black and Latino youth as well as adults (“racialized marketing”) [27–29]. F&B marketing targeted to the US Black population is multi-faceted with a long and mixed history that leverages structural racism, making it especially difficult to counter from a public health perspective [30]. Among Black youth, this targeting creates a triple jeopardy of exposure to marketing of unhealthy foods to general audiences, to youth in general, and to Black communities in general, reflecting the additive impact of overlapping systems of oppression and intersecting identities—referred to as intersectionality—in F&B marketing [31, 32]. Moreover, evidence suggests that Black adolescents are more responsive to some types of advertising and promotion from an identity-formation perspective and because Black celebrities who are prominent in many F&B ads are trusted and viewed as role models [33–35]. For example, a study of US advertisements from 1990 to 2017 found that sugar-sweetened beverages or products were the single largest category of brand endorsements for Black, male, and sports-related celebrities [36].

Here we report evaluation results for an online social marketing campaign developed and implemented by a multidisciplinary team of academic, advocacy, and advertising partners and branded “Operation Good Food & Beverages (OGF&B).” The campaign aimed to catalyze a change in the conversation about solutions to racialized targeted marketing of unhealthy foods and beverages, focusing on solutions. The core, underlying theme was an inspirational call to action by and for Black youth and others in or allied with Black communities for shifting toward more marketing of healthy relative to unhealthy foods generally and related to Black youth. Campaign elements included a website with relevant graphics and messages, social media accounts, and a signable petition to join the call to action. The current article describes the campaign rationale, development, and findings from the mixed-methods evaluation approach designed to answer questions about reach, audience engagement and factors related to participation.

## Background

The campaign brand and subtitle—Operation Good Food and Beverages (OGF&B): A Black Community Imperative—were intended to denote its grounding in Black organizations and sense of urgency for taking action on the need for healthier food in Black communities. A pilot study in which findings from Black adults, including caregivers of youth under age 18 years, suggested that seeing Black celebrities advertising healthy foods might sway opinions of Black youth who sought to emulate them [37]. Data describing the engagement of Black celebrities in F&B advertisements and the engagement of some celebrities during Michelle Obama’s *Let’s Move* campaign further motivated OGF&B as did evidence that many celebrities were speaking out on social or racial justice issues [36, 38].

**Fig. 1:**
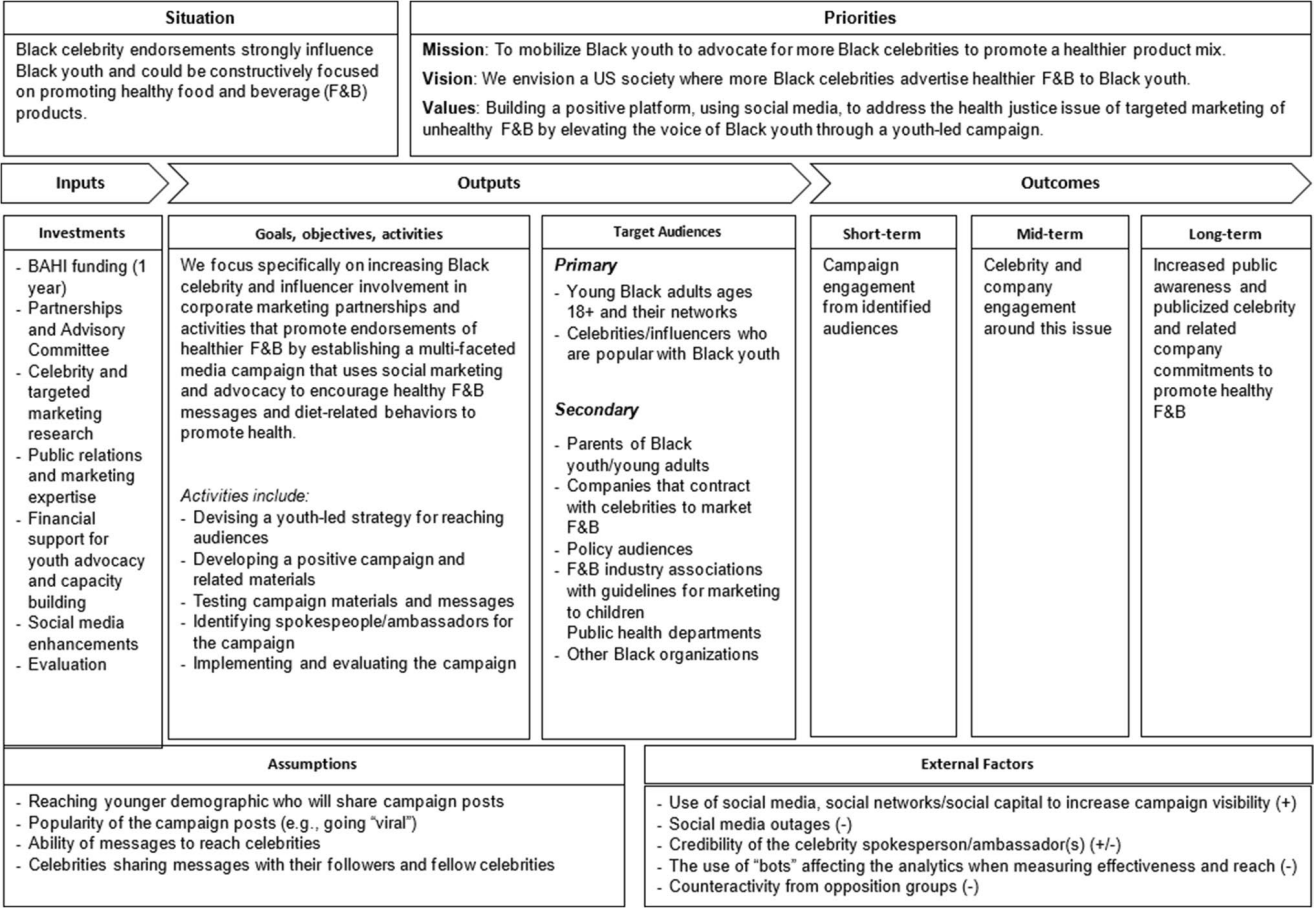
OGF&B theory of change framework—catalyzing a youth-driven public advocacy campaign to secure commitments from Black celebrities and influencers to endorse healthy foods and beverages (adapted from Kraak et al., 2022 [39])

As shown in Fig. 1, OGF&B’s theory of change was guided by an extensive, public health-oriented review of beverage campaigns [39]. The overall framing of the campaign and its messaging were positive and constructive, i.e., conveying what types of involvement and actions youth and adults want to see more of from popular opinion leaders in the Black community. In particular, Black celebrities, including both traditional music, film, and pop culture icons, as well as social media influencers, were a primary campaign audience in the hope that some would be persuaded to participate and endorse healthier rather than unhealthy products. Participating in a social media campaign typically involves engaging with and promoting content that aligns with campaign goals, include sharing posts, creating original content, and interacting with followers to raise awareness and drive action through personal social media accounts [39]. F&B companies contracting with Black celebrities for endorsements were also an indirect audience of interest. Desired short-term outcomes were increased awareness of the need for more marketing of healthy foods in Black communities. Celebrity and broader public and company engagement and participation were long-term outcomes.

## Methods

### Evaluation Approach

A multidisciplinary study team evaluated Operation Good Food & Beverage (OGF&B) including public health researchers from Drexel University (SA, SK, TE) who participated in the design and implementation of the online campaign, as well as team members with expertise in mixed methods program evaluations from the University of Pennsylvania (MK and MG—see the “Acknowledgements” section) who were not directly involved in OGF&B. The team reviewed and analyzed data collected from three sources: (1) online campaign and petition performance reports; (2) semi-structured interviews with campaign advisors and influencers; and (3) social media curated content from Instagram and TikTok. Analyses of these data were guided by the following study questions:

- RQ1: What was the estimated reach of the campaign?
- RQ2: What characteristics of social media posts drove audience engagement?
- RQ3: What factors were associated with participation in online campaigns in general and the OGF&B campaign specifically?
- RQ4: How did participants perceive the online campaign?

### OGF&B Development and Recruitment

Campaign development began in September 2021, and the campaign was active for four months, through the end of the 1-year funding period (May through August 2022). The campaign initially planned for social media advocacy as well as in-person enhancements. However, only the social media component was possible due to the extended duration of safety precautions associated with the COVID-19 pandemic. All communications and interactions related to campaign development and implementation were remote. Campaign creation, groundwork, infrastructure, and dissemination were directed and delivered on a *pro bono* basis by a third-party advertising agency whose portfolio was not related to the food sector. The advertising agency worked in close partnership with the team that was responsible for the campaign focus (SK and TE). Black high school and early college students (primarily members of the partnering youth leadership program) participated as paid consultants, and along with a master’s degree research fellow served as content advisors and creators from the earliest stages. The campaign was also informed by feedback and guidance from a panel of adult advisors with expertise in public health nutrition, marketing, and Black community health, and health advocacy.

The OGF&B web or digital device landing page featured images and text relevant to Black American culture to foster awareness of key food marketing issues and their relevance to Black community food and health justice. Links on the landing page pointed to OGF&B Instagram and TikTok accounts, prompts to sign the Change.Org petition, and to download the campaign social media toolkit (see Appendix A, OGF&B social media toolkit). Dissemination included organic social media posts and interactions as well as paid advertisements focusing on the New York City region for brief periods during the active campaign period. The advertising agency team engaged a talent agent who approached representatives of traditional Black celebrities after vetting for reputation and suitability as role models for Black youth. The agency staff directly approached other social media influencers after vetting on several characteristics, including alignment with the OGF&B content. Two TikTok influencers participated. Attempts to engage with traditional celebrities during the active campaign period were not successful. One partner, the Center for Science in the Public Interest, facilitated exploratory conversations with two of the authors (SK and TE) on behalf of the Council on Black Health and a colleague from the UConn Rudd Center with representatives of the National Restaurant Association, although these conversations did not lead to an ongoing dialog.

### Data Collection and Analysis

#### Online Campaign and Petition

From April 28 through September 1, 2022, the advertising agency collaborator launched and operated the multi-media OGF&B campaign including use of paid and unpaid/organic media content spanning both traditional media (e.g., press releases, online news articles) and new media (e.g., Instagram, TikTok, YouTube) to drive engaged audiences to the petition and website. On YouTube, for instance, OGF&B banners were added to YouTube videos in the New York region. The petition requested everyone to review and endorse the call to action urging restaurants serving Black communities to increase and promote healthy meal offerings and menu options to Black youth and teens. To monitor the progress across digital platforms, campaign metadata were collected that captured the total number of content impressions, website visits, and petition signatures. Impressions are a social media metric defined as unique views of content items (e.g., the number of times that an advertisement was seen on Instagram).

Specifically, the call to action encouraged the National Restaurant Association and its members, including independent and chain restaurant establishments, to adapt and apply the existing “Kids Live Well” [40] nutrition standards to their general menus, particularly for items that are popular and frequently purchased by Black youth up to 18 years old, and to amplify promotion of these options. Seeking parsimony in the campaign’s “ask” on food items relevant to Black teens, OGF&B leaders chose the National Restaurant Association, allowing the campaign to focus on well-documented reticence among industry segments to make health-related changes with the “Kids Live Well” initiatives for children over age 12. The OGF&B team did not choose potential alternatives (e.g., Children’s F&B Advertising Initiative) as targeting specific packaged F&B was hypothesized to be less effective due to various factors such as companies’ ability to substitute products across their large portfolios and dilute the visibility of targeted marketing patterns.

#### Social Media Content

Permanent public Instagram and TikTok content created by the OGF&B campaign accounts was collected that included text, imagery and videos, and social media metadata (e.g., likes, comments). A codebook was developed to characterize visual and thematic aspects of the social media content such as types of F&B portrayed, people being shown, content purpose (e.g., “Sign the Petition!”, event details), target audience, and constructs from Rosenstock’s Health Belief Model (e.g., perceived benefits of healthy diet, risk of exposure to unhealthy food advertising) [41]. Members of the study team (MK, MG, TE, SA) reviewed a sample of social media posts to ensure that all relevant aspects were captured through our codebook, and then coded the full dataset. We then examined how audience engagement varied by different aspects/types of social media posts.

#### Semi-Structured Interviews

A research assistant (MG) working with the project director for the evaluation (MK) conducted semi-structured interviews (*n* = 15), during the 3 months following the active campaign period. Potential interviewees were recruited using a list of potential contacts provided by the project director (SK). All participants provided informed consent and were compensated $50 for their time. Interviews engaged diverse stakeholders, including advisors, youth consultants, participating influencers, and influencers who chose not to participate. Interviews delved into dimensions of engagement with the OGF&B campaign and other similar initiatives, with a particular focus on understanding the nuances of decision-making processes regarding potential partnerships or collaborations. The interview guide (see Appendix B) encompassed a range of topics related to OGF&B, as well as participants’ general experiences with and perspectives on social media advocacy campaigns. Questions explored participants’ motivations, challenges, perceived impact of their involvement with OGF&B, and their engagement with other campaigns. To ensure relevance and appropriateness, questions were tailored for specific campaign roles and experiences of the different participant groups. For instance, youth consultants were asked about their contributions to content creation and message framing, while influencers were probed on their decision-making criteria for aligning with health-focused social media campaigns. Participation in the OGF&B campaign was defined broadly to capture a wide range of engagement levels and types. For those who did not engage in content posting on social media—particularly influencers and youth consultants—participation was characterized by their contributions to project design, development, and strategy formulation.

A codebook was developed to facilitate the analysis of interview data, using a grounded theory approach to ensure emerging themes were systematically identified and categorized [42]. The final codebook (see Appendix C) provided a comprehensive framework for analyzing the diverse insights shared by interviewees. All interviews were audio-recorded and transcribed verbatim. Identifying information was removed from transcripts prior to analysis. Transcripts were imported into NVivo software (QSR International) to facilitate coding and thematic analysis. Our thematic analysis was directed toward answering our study questions. We identified key themes iteratively throughout the coding process via group discussions, code summaries, and coding queries and comparisons.

## Results

### Online Campaign and Petition

Through the paid media content, such as Instagram, YouTube, and TikTok advertisements, the campaign garnered a total of 3,148,869 impressions. The campaign also received a total of 10,164 organic impressions from content posted on OGF&B’s Instagram and TikTok profiles. Both paid and organic media content items directed audiences to visit the OGF&B website, resulting in a total of 3,799 unique website visits (< 1% of impressions). Of users who visited the OGF&B website, a total of 1,077 signatures were collected for the campaign’s petition during the active grant period—nearly a third of website visitors (28%).

### Social Media Content

A total of 10 TikTok videos, 13 Instagram Reels (i.e., videos), and 21 Instagram posts were collected from OGF&B social media platforms. Through our analysis of the campaign’s social, we identified several factors associated with increased audience engagement (i.e., likes, views). Instagram Reels (i.e., videos) received significantly higher likes (*p* < 0.001) compared with static posts or TikTok videos, respectively 22.3 likes (SD = 11.6) per Reels video on Instagram versus 16.5 (SD = 6.3) for Instagram posts and 3.5 for TikTok videos (SD = 2.1). Content that featured people received significantly more likes compared to content without people, such as images of F&B items alone, respectively 19.6 likes versus 11.3 (*p* = 0.0129). Videos that featured people also received more likes compared to videos without people, albeit not significant statistically (*p* = 0.134), respectively 572.3 versus 238.4 views. Content that integrated elements of the Health Belief Model (e.g., benefits of healthy food, harms of poor diet) received 18.3 (SD = 4.7) likes on average compared with 14.4 (SD = 1.7) for content without (*p* = 0.343).

### Semi-Structured Interviews

A total of 15 individuals were interviewed between November 2022 and December 2022, including eight (8) adult campaign consultants, three (3) social media influencers, two (2) youth campaign consultants, and two (2) advertising agency collaborators. All participants were female, and the average age was 41 years old (SD = 11.3; median = 40). Interviews revealed that social media campaign participation—both with OGF&B specifically and online campaigns in general—is driven by a desire for alignment with personal values, relationship building, community contribution, personal growth, and ethical practices, while decisions to decline often stem from a lack of value exchange, ethical concerns, and conflicts with personal or organizational values. Below, we discuss the reasons identified from youth influencers and advisors for their participation in the OGF&B campaign. We then discuss reasons for participation in campaigns in general. Table 1 presents key decision-making factors for participating in OGF&B and general online campaigns.

**Table 1:** Decision-making factors for participating or not participating in OGF&B. (Note: Unless otherwise indicated, quotes are from people who participated directly in or advised the campaign)

Theme	Illustrative quotations related to OGF&B or when indicated, campaigns in general
Level of interest in the cause	*“When they reached out and told me what the initiative was, I couldn't help it. It was everything to me because child nutrition is what I really focus on.”* *“Is [a given project] going to be able to keep my interest? Because I don't know, my intention can kind of be like all over the place at times… I need to be interested throughout the way, even when it gets difficult or hard.” [General]*
Anticipated impact	*“The first thing – the first two things, and they're tied, and I will say impact and compensation. And when I say compensation, I don't necessarily mean money…the compensation was helping Black youth be healthy; that was payment enough for me.”* *“I think I’m considering their stake in the culture so as someone who is pro-Black influence really about helping the diaspora connect to the land. I really want to make sure the people I work with has some stake in our community and I feel like they have good intentions because a lot of people will just use me just to get in front of their target audience and not necessarily care about the issues that’s going on in the community.” [General. – Declined to participate]*
Degree of alignment with personal beliefs and values or mission	*“Well, the main thing is considering if it’s an alignment with where I want to go. Because for me, I want to make a better outlook of …youth, because people normally have a negative perception of youth…So like when I did, I decided to do OGF&B. It was like, Well, this is something new that’s going to kind of bring me outside of my shell, outside of my box. But at the same time, the work that we're doing this project is going to be impeccable…. And I feel like it is creating a better representation of [local] youth. So, I think about alignment.”* *“I want to feel like I’m aligned with values. I want to know the motive of the person that I’m collaborating with… I don't want to do anything that feeds those systems that cause harm to people.”* *“Mostly I’m thinking about whether or not the project is aligned with the mission that I have for the work that I’m doing, and whether or not I can be fairly compensated for the work that I do.” ​[General comment]* *“And what I factor in when I’m looking at different brands who either probably approach me or ones that I’m looking to get involved with is like, do they have a core mission that’s very strong? And do they have data as well as proof points that back up that mission? I think that’s important as well. You can have a nice talk, but if you don't actually put that into action, it can fall flat.” [General]*
Importance of compensation	*“Towards the end, after it was said and done, they did offer monetary compensation, but I consider one the impact I’m going to have. […] The compensation was helping black youth be healthy; that was payment enough for me.”* *“[OGF&B is] the kind of thing that I would do for free. Normally, the things that I get paid for I would do for free. Because my mentor, she says, ‘Do you think that you would do for free?’ […]. If it’s not something that you're truly passionate about, it’s easy to just be like, “Okay, well, let me throw in the towel.” But the things that I do, I normally would do for free. But getting paid is a bonus.”* *“If they're like hey can you do this for free? It'll be good exposure. It’s like I don't need necessarily exposure unless it’s a platform that I was like oh I want some insight into that space. I want to connect with people in that space. There has to be some type of value exchange a real value exchange and not just exposure because I can get that by myself that’s fine.”* *“For sure. Getting anybody to collaborate with anything you should pay people off… if you actually are looking for influencers to help push your agenda you have to be willing to compensate them.” [Declined to participate]*
Expectations and capacity	*“I think the big thing was probably time. It was probably like I didn't know how much I'd get to in that moment. It could have been either I’m working on another campaign, or I had something going on at work. That’s a busy season. I don't even honestly remember at what time frame, when it started because I’m always thinking about just everything, but yeah.” [General]* *“And, I guess, as far as prioritizing, I try to see what they do outside or what the timeline is and try to put things in chronological order. And also think of again impact if something is like quote-unquote, do, but it’s not really impacting anything, it’s something minor, then it won't get as much attention as something that’s big or front facing.”*
Relationship and community building	*“I do feel like I and others as well were hopeful that this effort would be led by teens and that it would have their real voice and stamp on it.”* *“And I think the Council on Black Health was an interesting opportunity for me to, as well as my team, to explore what that would entail within community-based organizations and grassroots type of approach, such as this Operation Good Food & Beverages campaign. And I think that’s part of why I was gravitating towards that. Is just that the betterment of people and equal opportunities when it comes to healthcare as well as to food.”* *“Anything that helps the underserved community is pretty much the bar and then based on whether or not I have experience and or relationships in those spaces from the corporate side matters as well because I do believe that sustainable change only happens when it’s all hands-on deck.”*
Professional development	*“However, I have recently become more active and engaged on social media because I would like to be an entrepreneur and I would like to get into social media marketing or just marketing in general or digital marketing.” [General]*
Ethical and financial conflicts	*“It was really difficult to find researchers who didn't have conflicts of interest or funding from the food industry.” “The first thing I consider is if there is any industry funding. And if there is I don't get involved in it. And the reason for that is it like – it becomes difficult to be objective in my opinion if there’s you know food industry funding behind the project.” [General]* *“We pretty much do not take any funding or collaborate with industry in that way because we want to be seen as impartial and that our research is really free from industry influence.” [General]* *“I had an academic who was being funded by a mainstream beverage company asked me to participate as an advisor. And I declined to do that because of who the funder was… I feel that the product causes harm generally and specifically in the Black and Brown community.” [General]*

#### OGF&B Participation

Youth social media influencers who did not participate in the OGF&B campaign gave reasons tied to their personal and professional priorities. One influencer highlighted a busy schedule and the importance of compensation, suggesting that the campaign did not align sufficiently with their current commitments or financial expectations. Similarly, another pointed out the significance of time constraints and the prioritization of paid opportunities over unpaid ones. Both emphasized the need for meaningful engagement and fair compensation, reflecting broader concerns within the creative community about equitable treatment and recognition of their work.

The main reasons youth and others decided to participate in the OGF&B campaign were rooted in their commitment to community welfare and personal alignment with the project’s goals. One influencer was driven by a professional focus on child nutrition and a desire to promote healthy eating habits among youth, valuing the campaign’s potential impact over financial gain or compensation. One youth participant also emphasized the importance of alignment with personal goals, such as improving the outlook for youth in their city and maintaining interest in the project. Both highlighted the significance of participating in initiatives that reflect their values and have the potential for meaningful impact, showing a thoughtful consideration of how their involvement would contribute to the broader goals of promoting health and well-being in their communities.

#### Campaigns in General

We identified a range of reasons why individuals chose to participate or declined to participate in social media campaigns. Participation in social media campaigns was described as driven by motives that align with their personal, social, and ethical values, such as supporting causes that resonate with their beliefs, fostering community connections, contributing to societal well-being, seeking personal growth, uplifting local economies, and promoting sustainability and ethical practices. A desire to enact positive change and maintain integrity by avoiding conflicts of interest also plays a significant role. Conversely, reasons for declining participation include the lack of fair compensation or value exchange, ethical concerns, misalignment with personal or organizational values, and initiatives that compromise their principles. This dichotomy highlights the importance of alignment with personal values, ethical considerations, and the pursuit of meaningful impact over superficial engagements, underscoring the nuanced decision-making process behind participating in or declining social media campaign opportunities.

#### OGF&B Perceptions

Participants described a range of key strengths of the OGF&B campaign including engaging with live events on Instagram, impactful visual content, effective collaboration, open communication, and strategic use of social media. The campaign’s collaborative efforts were commended for bringing together various stakeholders to focus on problematic targeted marketing to Black youth. Moreover, the campaign’s commitment to open internal communication was crucial for preempting and addressing potential issues among the multidisciplinary team effectively. Finally, the strategic use of platforms like TikTok and the successful engagement of influencers were instrumental in gaining organic traction, showcasing the campaign’s innovative and adaptable approach for reaching its target audience. Table 2 presents representative quotes of what participants considered as OGF&B’s strengths.

**Table 2:** Perceived campaign strengths identified through semi-structured interviews. (Note: Unless otherwise indicated, quotes are from people who participated directly in or advised the campaign)

Theme	Illustrative quotes
Engaging and relatable content	IG live sessions:* “And the IG live which was definitely my favorite part which I want to continue…So that was the best part.”* Videos and visual content:* “Some of the other videos they posted were really great. I love saying that the young Black man throwing up the vegetable, like I love. That was one of my favorites, favorite ones because you don't see that.”*
Collaborative efforts and partnership	Strong Partnerships:* “I think the team as a whole, both working collaboratively, I can't even talk, collaboratively with Council on Black Health, I think it was a strong partnership.”* Inclusive brainstorming and collaboration:* “And I think the folks at Council on Black Health did a really good job of bringing together a lot of folks who are working on this topic independently and at times coming together.”*
Effective communication and adaptability	Open Lines of Communication:* “Yeah. I think just overall that communication and like you would think that would be like a major thing that collaborators focus on but not all of them do but they really did focus on keeping the lines of communication open […]”* Adaptability in messaging:* “And then also to once they realized that they weren't the best messengers for the [youth members], they brought in a consultant who could better help carry the messages […]”*
Innovative use of platforms and influencers	Leveraging social media platforms: *“Having some nice organic traction, getting on a platform like TikTok where it’s a youth-based type of platform, which is very key to this organization in this campaign, was really influential.”*
Asset-based framing and positive messaging	*“I think they really wanted to do something different build and be very positive in the way that the messaging was positioned, and I say that so more asset-based more, so not coming at it from this deficit approach, but really asset focus […]”*

Many suggestions were made for improving campaign effectiveness. As shown in Table 3, interviewees emphasized the need for consistency and preparedness in content creation and advocating for a more systematic approach to minimize gaps between posts. They acknowledged the benefits of having a bulk of content ready for scheduled release. The importance of in-person engagement was highlighted, especially post-pandemic, to foster authentic connections and showcase real interactions with target audiences. Streamlining the campaign’s focus was also suggested, making the mission and call-to-action clear and specific to enhance audience understanding and engagement. Enhancing social media engagement was another key area, with recommendations for adding a personal voice to the campaign (e.g., a spokesperson or ambassador), particularly on platforms like TikTok, to improve engagement and accessibility. Long-term planning and securing multi-year funding were advised to allow for sustained campaign impact and progression. The use of incentives for influencers and maintaining a consistent content cadence on social media were emphasized for sustaining impact and building on initial rapport. Additionally, fostering curiosity and understanding between public health and industry stakeholders was seen as crucial for successful collaboration and addressing any misalignments. These suggestions collectively aimed at increasing the effectiveness, reach, and impact of campaigns.

**Table 3:** Areas of improvement and recommendations for OGF&B

Area of improvement	Recommendation(s)	Illustrative quote(s)
Content scheduling and preparation	• Develop a content calendar to ensure a consistent posting schedule • Create content in bulk to avoid lapses between posts and maintain audience engagement • Utilize tools for automated posting to manage content dissemination effectively	*“I do like the fact that they already had some posts put together. I’m going to try to come up with things more like a bulk of things at once so that I can already have them to roll out like on a time schedule. It was just that there’s so much going on, I had to go out of town I think right around that time and so… Just to be more prepared to do more to give more out. So, there’s not so much lapse between posts.”*
Engagement and outreach strategies	• Increase in-person engagements and community involvement to add a dynamic layer of interaction • Leverage influencers and community figures to enhance outreach and relatability • Explore interactive content formats and platforms, such as TikTok, for higher engagement	*“I’m going out in these places and talking to these youth and team in person and posting that because that’s a whole ‘nother dynamic, because it has the body language involved… Like really – I want to see people interact with the people we're trying to reach, go into the communities and put that out there, and have that showcase.”* *“I think it needs to have a voice behind it and to be able to better connect with their target audience because I don't think there’s a lot of engagement.”*
Target audience clarity and message simplification	• Clarify the target audience and tailor the campaign’s messaging to their specific needs and interests • Simplify the campaign’s call to action to make participation clear and straightforward • Ensure the campaign’s objectives are easily understood and actionable	*“Effective campaigns are easily understood. They are very specific in very many ways, both in terms of the audience they're trying to reach and the message they're trying to impart, and they are very specific and they're actionable.” –*
Diversity and representation	• Ensure diverse representation within the team reaching out to influencers and communities, particularly those from Black or people of color backgrounds • Foster leadership opportunities within campaigns for youth, allowing them to guide messaging and outreach strategies • Recognize and address the importance of identity in the campaign’s work, focusing on specific communities	*“On your team, are there Black or people of color?… Okay. Are there Black people reaching out to these other Black influencers to discuss these things with them?” – [Declined to participate]*
Influencer collaboration and compensation	• Prioritize securing and compensating influencers to ensure their genuine involvement and support for the campaign • Develop strategies for maintaining and expanding relationships with influencers to amplify the campaign’s reach	*“It’s very hard to secure influencers these days from that pro bono perspective and them doing or getting involved purely from that altruistic standpoint… These days on social, it’s all paid to play.”*
Long-term planning and funding considerations	• Explore diverse funding sources, including grants, partnerships, and industry collaborations, to ensure long-term viability • Develop a clear value proposition for potential funders and collaborators that highlights the mutual benefits of supporting the campaign	*“I think that’s the problem with funding, right? Sometimes you get funding for just a year and then you're only, you have to be realistic in what you can deliver in a year.”*
Curiosity and understanding for collaboration	• Adopt a stance of curiosity toward different stakeholders to better understand their perspectives and motivations • Facilitate open dialog between public health advocates and industry representatives to find common ground and mutually beneficial solutions	*“We have to be more curious about each other so that we can learn and then find places to meet in the middle. […] You have to be curious about what the other entity is doing or how they’re making decisions. [For example] this guy’s company is literally, it is just a marketing company. It’s the same as Coke, Pepsi but they're all the same, but his product is absolutely fully 100% aligned with public health goals. But no one has reached out to them.”*

## Discussion

OGF&B resulted in modest success in the face of challenges posed by COVID-19 pandemic restrictions and a short funding period that limited the duration of the active implementation. The campaign leveraged communication channels including traditional media (e.g., press releases, online news articles) and new media (e.g., Instagram, TikTok) to reach more than three million viewers. The millions of impressions, robust website activity and substantial follow through to signing the petition suggest that the campaign was engaging and relatable, resonating with the demographic groups targeted by the advertisements. Results point to key strengths that contributed significantly to OGF&B’s impact and reach, e.g., comprehensive approach, integrating content development, stakeholder collaboration, and innovative use of social media, emphasizing engagement, adaptability, and a clear focus on the target audience’s values and preferences, ultimately mirroring the characteristics of other successful campaigns like San Francisco’s *The Bigger Picture* [43, 44] and Seattle’s *Be Ready Be Hydrated* [45, 46].

Our evaluation of social media content identified features associated with increased audience engagement, a key factor necessary to translate impressions to site visits, and consequently, site visits to petition signatures. These results are pivotal for understanding and optimizing future social media campaign strategies. Firstly, the preference for Instagram Reels over static posts or TikTok videos (“TikToks”) highlights the effectiveness of certain platforms and content types in engaging audiences. This insight is crucial as it suggests that dynamic video content, particularly on Instagram, resonates more strongly with users than other formats. Importantly, Instagram Reels and TikToks both represent two sides of the same coin—that is, the tall video format—and while there is cross-pollination of content vis-à-vis sharing and linked accounts, campaign leaders should seek to maximize reach across both platforms effectively and efficiently. For example, available usage data indicate that four in five TikTok users are also active on Instagram, whereas slightly more than half of Instagram users are also on TikTok [47]. Instagram Reels are part of the broader Instagram ecosystem, allowing users to leverage their existing follower base and integrate content with Instagram Stories and posts. TikTok, on the other hand, is a standalone platform with a younger user base and a robust discovery algorithm that can potentially reach a wider audience through the “For You” page. These distinctions can influence engagement, as Instagram Reels may benefit from integration within Instagram, while TikTok’s algorithm-driven content distribution may provide broader initial exposure to diverse audiences. Therefore, in the Venn Diagram of Instagram and TikTok influencers, consider targeting those at the center who have mastered both platforms.

Content featuring people receiving more likes than images of inanimate objects alone, like F&B photos, underscores the importance of incorporating a human element. Social media content featuring people has previously been shown to captivate online audiences and garner more likes, more comments, and more shares—key engagement metrics—such as promoting HPV vaccination on Instagram [48, 49] or PrEP on Twitter [50]. Posts featuring people likely create a more engaging and transporting experience with which audiences identify, emphasizing the power of narrative persuasion via personal stories and testimonials in making content compelling [51, 52].

Our findings also suggest that audiences are drawn to videos that seem more authentic and engaging, which is vital for campaigns looking to maximize reach and impact through video content. In OGF&B, videos featuring people garnered significantly more views than those without people, such as videos with F&B products, reiterating the importance of personalization and human connection in video content. Moreover, the increased engagement with content that incorporates educational elements—as we observed with Rosenstock’s Health Belief Model [41]—indicates that audiences appreciate content that provides value through information and education. Prior research supports that informational aspects are particularly relevant in health and well-being campaigns, where educating the audience can be as crucial as engaging them [53, 54]. These insights collectively offer valuable guidance for tailoring social media strategies, ensuring that campaigns not only reach but also resonate with their intended audiences, thereby maximizing their effectiveness and impact.

OGF&B’s approach to fostering healthier food choices within Black communities builds on prior initiatives focusing on positive, asset-based messaging and community empowerment to engage its target audience effectively [39]. For example, the City of Seattle launched *Be Ready Be Hydrated* as a bilingual, community-informed campaign focused on countering marketing of sugary beverages to Black and Brown youth by emphasizing the well-being and positivity of these groups [45]. With direct input from local youth, *Be Ready Be Hydrated* employed a range of digital strategies to achieve nearly 5.9 million impressions [46]. In response to the challenges of 2020, including the COVID-19 pandemic and social justice movements, *Be Ready Be Hydrated* adapted messaging and content to maintain engagement and support across virtual platforms. In the San Francisco Bay Area, *The Bigger Picture* campaign took a broader socioecological approach to tackle type 2 diabetes among minority and low-income youth [43, 44]. *The Bigger Picture*’s goals were to increase public health literacy, changing social norms, and encouraging civic engagement by involving youth in the creation and dissemination of content that resonates with their experiences and perspectives. The campaign employed youth-generated spoken-word messages to address the broader environmental and social determinants of health contributing to the diabetes epidemic, and the multimedia campaign was complemented by health education in public high schools.

Like *The Bigger Picture* and *Be Ready Be Hydrated*, full implementation of OGF&B has the potential for unique contributions to public health initiatives within youth as vulnerable to adverse effects of marketing practices, all while emphasizing the importance of youth involvement in content development. OGF&B sought to tap into cultural relevance, celebrity, and influence to generate demand for healthier F&B alternatives. *The Bigger Picture* distinguishes itself through a youth-*led* approach, allowing young voices to authentically highlight the socioecological factors behind type 2 diabetes [43, 44]. On the other hand, *Be Ready Be Hydrated* employed a community-centric strategy, emphasizing grassroots efforts and leveraging digital platforms for broad outreach [45, 46]. Direct engagement with youth not only informs content creation but also ensures that the messaging resonates with the intended audience. Without COVID-19 limitations on the potential for in-person activities, OGF&B could have benefited from a multi-pronged strategy that included online and social media content as well as community engagement events.

Beyond targeting nutrition education and dietary behaviors, public health campaigns have long utilized similar community engagement methods to OGF&B. For example, Howard County’s *Better Choices, Better Health* initiative was a comprehensive public health campaign designed to improve overall community health through policy changes, educational programs, and community engagement [55]. Targeting a wide demographic, *Better Choices, Better Health* focused on multiple health behaviors: increasing physical activity, promoting nutritious eating, and implementing policies that create healthier environments. Key campaign components include improving access to healthy foods, creating safe spaces for physical activity, and educating residents on making healthier lifestyle choices. In contrast, OGF&B specifically targets Black communities, leveraging cultural relevance, influencer involvement, and youth involvement to generate demand for healthier food and beverage alternatives.

Using a similar approach for a different health issue, *PrEP4Love* was a City of Chicago sex-positive public health campaign aimed at increasing awareness and uptake of pre-exposure prophylaxis (PrEP) for HIV prevention among Black sexual minority men and transgender women [56, 57]. *PrEP4Love* utilized culturally responsive and inclusive messaging to address stigma and promote sexual health. Like OGF&B, *PrEP4Love* leveraged community input and media strategies to resonate with its target audience but focuses on reducing HIV transmission rather than dietary habits. Together, these campaigns showcase diverse yet complementary methods of engaging communities and promoting health, highlighting the critical role of tailored strategies in addressing public health challenges.

### Implications for Future Campaigns

Based on the responses about motivations for participation or non-participation in certain campaigns, we can derive key strategies for encouraging engagement. First is the alignment of a campaign with personal values and interests. People are significantly more inclined to participate in initiatives that resonate with their beliefs, passions, and life experiences. A personal connection not only motivates initial involvement but also sustains long-term engagement, as participants reported feeling a deep sense of purpose and fulfillment. Campaigns that clearly articulate their alignment with potential contributors’ values can thus tap into a powerful motivational force. Moreover, projects that offer opportunities for personal and professional growth, or that contribute positively to community well-being, are particularly attractive. These aspects address an intrinsic desire for self-improvement and societal contribution, making participation not just a duty, but a rewarding experience.

Another critical factor is the perceived value and compensation associated with participation. While not all campaigns can offer financial rewards, recognizing and valuing participants’ time and contributions is essential. This can be achieved through various forms of acknowledgment, such as public recognition, opportunities for skill development, or networking prospects. Conversely, a lack of clear value exchange, such as projects perceived as irrelevant or not offering any tangible benefits, can lead to declined participation. Ethical considerations also play a significant role. We learned that individuals are more cautious associating with campaigns that might conflict with their ethical standards or involve questionable practices, highlighting the importance of transparency, integrity, and ethical conduct in campaign management. Last, despite OGF&B’s successes, we observed a clear absence of direct mentions of Black issues in some interviews, pointing toward an area for improvement in future campaign designs. For example, the focus on diet and nutrition could have been enhanced by integrating content reflecting ongoing national discourse about health inequities and their causes. Engaging more deeply with the specific concerns and experiences of Black communities could enhance the relevance and impact of such initiatives. By addressing these key areas—value alignment, personal and community benefit, recognition of contribution, and ethical integrity—campaign organizers can significantly enhance participation rates and foster a committed and enthusiastic supporter base.

The substantial donations of creative and technical content for developing and implementing the campaign effectively doubled the resources and elevated the expertise available, which must be acknowledged when considering what OGF&B accomplished. With this in mind, the campaign’s impact—especially in terms of creating a platform for direct engagement and its positive messaging framework—adds valuable insights to the ongoing discourse on effective strategies for countering targeted marketing of unhealthy F&B to youth populations or minority communities. That the campaign was active for only four months and took place during the pandemic were major limitations, the latter rendering it impossible to include direct in-person engagement to prime and support the online activity. Pandemic restrictions had a major adverse influence on the efficiency of campaign development among, and in some cases within, collaborating organizations. Future campaigns could improve on these limitations by better engaging celebrities and influencers, expanding communication channels (e.g., television, newspaper), and establishing a consistent online presence. Finally, the campaign’s heavy reliance on social media platforms for dissemination and engagement introduced a selection bias by inherently excluding individuals and communities with limited internet access or those who are not active users on these platforms.

## Conclusion

The landscape of F&B marketing has become a focal point of public health advocacy, highlighting the stark misalignment between marketed products and the nutritional guidelines that aim to safeguard health. Across the USA and around the globe, growing concern underscores the urgency of addressing the pervasive influence of marketing strategies that prioritize profit over public health, particularly those targeting youth. Among the most affected are youth in Black and Latino communities, who are disproportionately exposed to advertisements for unhealthy F&B options. Marketing and advertising exposures contribute not only to immediate health risks but also to the long-term burden of chronic diseases. The critical need for a paradigm shift in marketing practices is evident, calling for a comprehensive approach that encompasses regulatory changes, community empowerment, and the promotion of healthier alternatives. It is within this broader struggle against harmful marketing practices that we must contextualize OGF&B and its novel campaign targeting Black communities. In doing so, we may better appreciate OGF&B’s significance as not merely a social media campaign – but as part of a larger social movement toward rectifying the deep-seated inequities in food marketing and consumption patterns.

In conclusion, this evaluation of OGF&B bears significant implications for health equity, advocacy, policy change, and the strategic use of media campaigns to address complex health issues. By actively engaging communities, influencers, and policymakers, the project lays the foundation for a healthier environment and future for Black youth and Black communities. To our knowledge, this was the first youth-engaged health justice movement explicitly designed to address Black celebrities’ and influencers’ endorsements of F&B products. Recognizing the profound influence these public figures wield in shaping attitudes and behaviors, particularly among Black youth, OGF&B aims to redirect this influence toward promoting healthier choices through empowering youth to lead efforts in reshaping societal perceptions and promoting health-conscious decisions. OGF&B holds potential implications for policy recommendations and changes, perhaps most importantly the need for a proactive approach that influences systemic factors contributing to childhood obesity coupled with evidence-based policy to reshape industry practices related to F&B marketing to children.

## Data Availability

Data supporting the findings of this study are available upon reasonable request from the corresponding author (MK).

## Appendices

### Appendix B. Semi-structured interview guide

#### Section 1: Background

***PROMPT: Thank you. I’d like to begin with discussing your background and what led you to working with the campaign***.

1. Tell me about what you do outside of the campaign.
  *Probe: When – years, age*
  *Probe: Platform(s)*
  *Probe: Personally or professionally*
2. What led you to start working as an advisor for Operation Good Food and Beverage?
  *Probe: Important experiences (e.g., campaign, viral post)*
  *Probe: Opportunity for paid marketing work—$$$*
  *Probe: Life events*
3. To what extent have you been involved with similar campaigns?
  *Probe: Social media?*
  *Probe: Causes beyond healthy food and beverages*

#### Section 2: Social media collaborations

***PROMPT: Great, now I’d like to talk about how you consider different types of social media opportunities*** *.*

4. When you’re approached with an opportunity to collaborate someone or something, such as an organization or company, social media, what types of things do you consider when thinking about working with them?
  *Probe: Motivating factors – i.e., facilitators*
  *Probe: Conflicts of interest*
  *Probe: Competing priorities*
  *Probe: Time, $$*
  *Probe: Beliefs about someone/something*
5. Could you describe an example of a time when you declined an opportunity for a social media collaboration?
  *IF NO: What opportunities would you decline?*
  *IF YES: What led you to supporting that cause?*
6. How do you decide what types of causes to express support or advocate for on social media?
  *Probe: Paid versus unpaid work*
  *Probe: Personal experiences (e.g., DV/IPV)*
  *Probe: Identity-based (e.g., LGBTQ+)*
  *Probe: Beliefs about cause*
7. Could you describe an example of a time you expressed support or advocated for a cause on social media?
  *IF NO: What cause or causes could you see yourself supporting?*
  *IF YES: What led you to supporting that cause?*

#### Section 3: Reflections on the Campaign

8. What was your level of involvement with the campaign?
  *Probe: Planning/development*
  *Probe: Execution and implementation*
  *Probe: Sharing posts*
9. Were there any pieces of that campaign that stood out particularly impactful? Explain.
10. How would you improve the campaign in the future?

#### Section 4: Beliefs and Values

***PROMPT: Okay, for my last few questions today I’d like to dive a little deeper into what influences someone’s decision to collaborate on social media*** *.*

11. What value or values are important to you in your work on youth-oriented campaigns?
  *Probe: Morality, ethics – right versus wrong*
  *Probe: Religious beliefs*
  *Probe: Political beliefs*
12. Could you describe an example of a time when you had a conflict between your values and a collaborators values?
  *IF NO: What types of conflicts do you think could arise, even if they haven’t yet?*
  *IF YES: How did that effect your work together?*

***For participants in campaign:***

13. What was your main reason or reasons for participating in the campaign?
14. What drew you into the campaign?
  *Probe: alignment with values*
  *Probe: alignment with brand*
15. What was your level of involvement with the campaign?
16. How would you improve the campaign in the future?

***For non-participants in campaign:***

17. What was your main reason or reasons for not participating in the campaign?

*Probe: misalignment with brand*

*Probe: financial*

*Probe: priorities (paid first before unpaid posts)*

*Probe: competing interests*

#### Section 5: Conclusion

***PROMPT: That concludes the formal questions I have for you today***

18. Do you have any final thoughts about anything we discussed today?
19. Is there anything that we didn’t discuss that you think we should know based on our discussion today?
20. Do you have any questions for me?

***PROMPT: Wonderful, thank you. This has been a productive and informative conversation and I very much appreciate you taking the time to speak with me. If you have any questions or thoughts after this discussion, please do not hesitate to reach out to us at [PHONE/EMAIL]. Thank you very much and enjoy the rest of your day.***

### Appendix C. Thematic analysis codebook

**Table:**

Code name	Definition
Social media use	Subcode to relevant valences as participants discuss social media use
Personal	Social media usage that is not related to work or another organization
Professional	Professional social media use on an account representing the participant
Organizational	Social media account on behalf of an organization
Relationship to campaign	Code to categorize patient in the listed roles: Youth Advisor, Campaign Advisor Influencer (Participating), Influencer (Nonparticipating), Industry Liaison, Content Creator, Other
Mission alignment	This code should be used when participants indicate that a collaboration/intervention/job aligns with values or priorities, either theirs or those of entities that they represent
Compensation	Apply code for any discussion of compensation/money
Campaign contribution	Subcode when participant identifies the work they did for the campaign
Development	Advisory meetings, background research, identifying potential partners
Implementation	Creating social media posts, running accounts, interacting directly with audience
Declined opportunities	Code when participant describes instances where they rejected or declined an opportunity
Motivation	Code when interviewee discusses why they choose to do something
Intuition/emotion	Code when participant mentions that they took an action because it “felt right” or they invoke an emotional connection
Decision-making	Apply when participant discusses how they make choices about collaborators, projects, etc
Personal brand	Use code when participant mentions cultivating or working in alignment with a personal brand or persona
“Valued”	Use when interviewee discusses the importance of feeling valued or being treated well in a professional collaboration/relationship
“Black youth representation”	Code any mention of black youth voices/representation
Industry collaboration	This code should be used whenever a participant mentions working with/alongside the food and beverage industry as a part of the campaign
Reflections/advice	Refers to any comments made about the program’s efficacy or recommendations for future iterations
Time/priority	Code when participant mentions how they prioritize work, the timeline of the study, or any general comments about having enough time/lacking time to do things
